# Customized 2D Barcode Sensing for Anti-Counterfeiting Application in Smart IoT with Fast Encoding and Information Hiding

**DOI:** 10.3390/s20174926

**Published:** 2020-08-31

**Authors:** Rongjun Chen, Yongxing Yu, Jiangtao Chen, Yongbin Zhong, Huimin Zhao, Amir Hussain, Hong-Zhou Tan

**Affiliations:** 1 School of Computer Science, Guangdong Polytechnic Normal University, Guangzhou 510665, China; chenrongjun@gpnu.edu.cn (R.C.); permanyu@126.com (Y.Y.); 2 School of Electrics and Information Technology, Sun Yat-sen University, Guangzhou 510006, China; chjiangt@mail2.sysu.edu.cn (J.C.); zhongyb7@mail2.sysu.edu.cn (Y.Z.); issthz@mail.sysu.edu.cn (H.-Z.T.); 3 School of Computing, Edinburgh Napier University, Edinburgh EH10 5DT, UK; A.Hussain@napier.ac.uk

**Keywords:** anti-counterfeiting, 2D barcode, information hiding, perceptual quality

## Abstract

With the development of commodity economy, the emergence of fake and shoddy products has seriously harmed the interests of consumers and enterprises. To tackle this challenge, customized 2D barcode is proposed to satisfy the requirements of the enterprise anti-counterfeiting certification. Based on information hiding technology, the proposed approach can solve these challenging problems and provide a low-cost, difficult to forge, and easy to identify solution, while achieving the function of conventional 2D barcodes. By weighting between the perceptual quality and decoding robustness in sensing recognition, the customized 2D barcode can maintain a better aesthetic appearance for anti-counterfeiting and achieve fast encoding. A new picture-embedding scheme was designed to consider 2D barcode, within a unit image block as a basic encoding unit, where the 2D barcode finder patterns were embedded after encoding. Experimental results demonstrated that the proposed customized barcode could provide better encoding characteristics, while maintaining better decoding robustness than several state-of-the-art methods. Additionally, as a closed source 2D barcode that could be visually anti-counterfeit, the customized 2D barcode could effectively prevent counterfeiting that replicate physical labels. Benefitting from the high-security, high information capacity, and low-cost, the proposed customized 2D barcode with sensing recognition scheme provide a highly practical, valuable in terms of marketing, and anti-counterfeiting traceable solution for future smart IoT applications.

## 1. Introduction

In today’s commodity era, merchants have fully realized the importance of brand effect, with the improvement of people’s consumption level. However, due to increasingly serious counterfeiting activities, the adoption of anti-counterfeiting technology has attracted widespread attention by enterprises of famous brand products in various countries. [1,2]. Among them, many conventional anti-counterfeiting technologies, such as laser anti-counterfeiting, ink anti-counterfeiting, printing anti-counterfeiting, and RFID anti-counterfeiting are widely used [3,4,5,6,7]. However, these technologies have the disadvantages of high cost, easy imitation, inconvenient detection, and lack of uniqueness. As an emerging industry, the Internet of Things (IoT) has become a new engine of the future economy and will profoundly change our future lifestyle. The basis for realizing the IoT is to attach a specific mark to each item, so a large number of marks are needed to achieve precise interaction and management. Common identifications mainly include RFID tags and barcode image identifications. The cost of RFID tags is declining, but it is still very high, compared to barcode image identifications. Although RFID tags can be identified clearly, they are susceptible to signal interference and environmental influences. As an essential entrance to the Internet of Things, two-dimensional (2D) barcodes are more deeply connected to people’s lives, because of low cost, convenient portability and sharing, and high recognition rate [8]. In financial payment [9,10], anti-counterfeiting authentication [11,12], electronic bills [13], and other smart IoT applications [14,15,16] are becoming more widely used. Meanwhile, anti-counterfeiting methods based on the application of 2D barcode sensing technology have become a research hotspot to solve the above problems.

**Figure 1:**
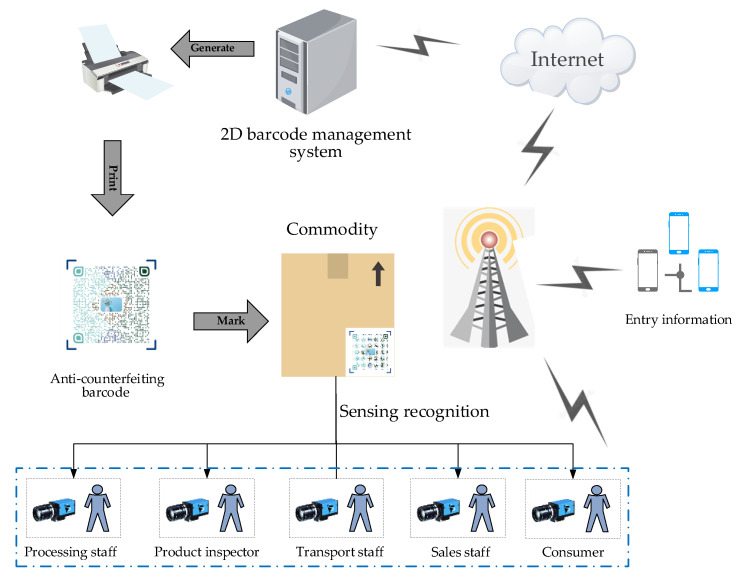
Anti-counterfeiting traceability system topology with 2D barcode sensing recognition.

Furthermore, the 2D barcode anti-counterfeiting label carries more irreplaceable functions with the development of technology, especially in brand marketing. Applying 2D barcodes sensing technology has become increasingly interesting in achieving distinctive marketing propaganda. In the anti-counterfeiting traceability smart IoT application, the process of obtaining brand-related information by scanning the anti-counterfeiting 2D barcode on the commodity is shown in Figure 1. First, the 2D barcode sensing anti-counterfeiting system enters the relevant information of products in different stages through the network, then prints out the anti-counterfeiting barcode attached to the corresponding commodity, while synchronizing the commodity and the corresponding 2D barcode information to the management system. All people in circulation can scan the anti-counterfeit barcode by a camera using a recognition software, to obtain the corresponding information of the product. In the traceability system, the anti-counterfeiting barcode identification is unique, which means that the relationship between the identification and the traced object is a one-to-one correspondence. The history of the product can be traced through the recorded identification. Moreover, traceability includes two aspects, tracking and tracing. Tracking is to follow the positive circulation process of the product, while tracing is to trace back the reverse circulation process of the product. In this way, it is ensured that when the product has problems, it can quickly locate the related problem link and solve it in time.

However, conventional 2D barcodes are only black and white images, which are not useful in aesthetics and data information security. It is necessary and exciting to design a kind of 2D barcode that visually reflects anti-counterfeiting and attracts people’s visual attention [17,18,19] to realize brand marketing. In recent years, high-security QR codes with closed-source technology are gradually becoming a hot topic for scholars. Consequently, we study a customized beautified barcode to improve the function of data security and anti-counterfeiting, which can not only realize the function of traditional 2D barcode but can also have better aesthetics to achieve the characteristic brand marketing.

In the beautified 2D barcodes methods, one is the fault-tolerant design based on itself, which embeds a picture by losing part of the error correction performance. Another method is based on information hiding that redefines the bit value by changing the pixels of the picture. The operation of embedding the image is directly applied to each module, and the modified module needs to be evaluated to ensure the decodability of 2D barcodes. Typical visual 2D barcode encoding methods are as follows:

Baharav [20] proposed an image embedding algorithm that combines image color with black-and-white modules. Chu [21] proposed a halftone QR code that encodes a series of binary halftone patterns rather than the QR code module. Then, the original image block is replaced with a halftone pattern based on the principle of maximum similarity. Its image embedding scheme is backward compatible with the scheme proposed by Visualead [22,23], which only sets the center pixel of the module to black or white, according to the encoded bits, and replaces the pixels around the module with the content of the embedded image. However, this type of embedded algorithms have different degrees of black and white dots in the beautified 2D barcodes, severely damaging the original picture quality and affecting the aesthetics of the visualized 2D barcodes. Samretwit et al. [24] further combined the 2D barcode correction level to find a region suitable for embedding the image. Such algorithms embed pictures by losing a portion of the error correction performance. Moreover, the embedded area should be carefully chosen to ensure the decoding robustness.

A recently proposed scheme to beautify QR codes, called QR Image [25], only modulated the central pixels of each module. It used some halftone masks to select the pixels outside each module and the center pixels to be modified for encoding to balance visibility and decodability. However, there is a problem with these methods. It is assumed that black and white pixels are a consistent number in each local window. This assumption is also applied to many current binarization methods [26,27], but the assumption is not valid.

The latest methods of studying image hiding information are to judge the pixel values between modules. Lee and Tsai [28] proposed an image hiding algorithm based on local image block binarization, which gives the image block a unified pixel value, by counting each image block average values of high and low pixel values. Lee et al. [29] proposed a new type of image 2D barcode, using the original QR code and then embedded it with images. However, the degree of aesthetics is insufficient because of the characteristics of the QR code. Chen et al. [30] first proposed a novel aesthetic 2D barcode—PiCode, which determines the encoding bits through the internal and external pixel difference between each module. Although the various performance indicators were improved compared to the previous methods, it requires more complex encoding processes and large amount of computation, so the encoding time and the corresponding decoding time is longer. Then, Lee et al. [31] proposed an improved visualized 2D barcode—RA Code, with more robust decoding ability and better aesthetic appearance, but the encoding time remains relatively slow.

Based on the analysis above, we concluded that the existing image embedding algorithms were more destructive to the carrier image, while the current beautified schemes could not optimize the human vision well, which cannot play a functional role in visual anti-counterfeiting. Additionally, an excellent 2D barcode identification, its encoding and decoding speed should be as fast as possible in actual application scenarios. Only in this way, can this image identification be convenient in propagating and sharing. Therefore, we propose a customized picture 2D barcode based on information hiding technology. By extracting the color channel information of the carrier image and encoding the information length, we adjusted the size of the carrier image and divided the sub-modules. Then, we determined the bit values of the sub-modules according to a specific sorting method, which improved the encoding and decoding speed. At the same time, the quality of the encoded carrier image was enhanced by the zeroing process. Intensive experiments demonstrated that the changed image quality was better than that of the above methods, which have strong practicality.

The innovations of the customized 2D barcode, compared to the existing 2D barcodes, based on information hiding technology, are summarized as follows. On the encoding side, an image encoding algorithm for visual 2D barcodes is proposed, based on Lee and Tsai [28] and Chen et al. [30]. Its main advantage includes improved encoded image quality and high efficiency. Compared to some existing methods of beautified 2D barcodes, the encoding speed was much faster, and image quality and encode indicators were preferable to those methods. Furthermore, the proposed encoding scheme was designed, based on the information hiding technology; hence, the anti-counterfeiting information could be hidden in the carrier images through encryption, which is difficult to copy.

On the decoding side, the sensing recognition sampling network was optimized to make the algorithm more suitable to the complex environment, thus, it could improve the decoding robustness, referring to the algorithms’ ability to cope with complex environments. Additionally, the proposed sensing recognition scheme was slightly better than the existing methods in bit error probability and decoding time, thereby achieving a good balance between aesthetics and robustness.

The remaining structures of the paper were as follows. Section 2 states the encoding process of the customized 2D barcode. Section 3 illustrates the details recognition process of the customized 2D barcode. Section 4 compares the customized barcode with several picture barcodes in perceptual quality, decoding time, and robustness. We also analyzed the extended performance under different testing conditions, to reflect the effect of the customized barcode. Section 5 concludes the customized 2D barcode scheme.

## 2. The Encoding Algorithm of Customized 2D Barcodes

In this section, the encoding process of customized two-dimensional (2D) barcodes are described in detail. We illustrate the modulation scheme of how the customized 2D barcode preserved image visualization, while improving the encoding speed.

**Figure 2:**
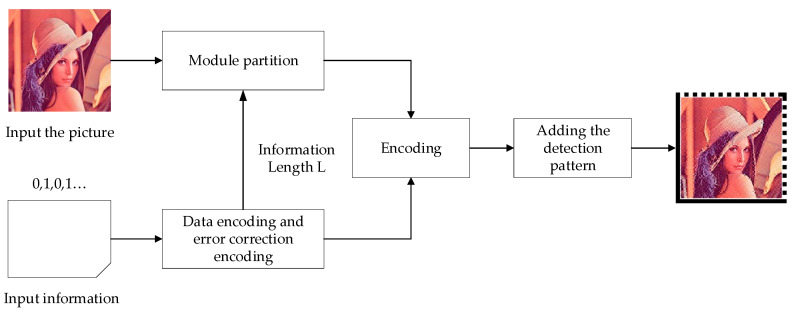
Customized 2D barcode encoding.

As shown in Figure 2, the encoding process was mainly divided into the following four steps.

1. Perform data and error correction encoding, based on the RS error correction coding mechanism to form a bitstream *B*, and obtain the information length *L*;
2. Input the picture and divide it into modules according to the information length *L*;
3. Encode the image based on encoding rules to obtain a picture-embedding 2D barcode;
4. Add the detection pattern according to the size of the formed code map, then form a complete customized 2D barcode.

Since the encoded target image is a color image, we choose the YCbCr channels to maintain color information consistency, extracting the Y channel of the YCbCr channels as the encoding object, to maintain the invariance of the CbCr channels. It is conducive to the improvement of image visibility. The customized 2D barcode system is mainly to study the color channels of the chromatic image, block algorithm of the unit image, and the detailed image encoding method. Encoded images are mainly used for testing with Lena image, and other images can also be encoded to suit brand anti-counterfeiting and marketing requirements. Additionally, the image resolution should be retained at an appropriate level to maintain picture visibility and the encoding robustness.

**Figure 3:**
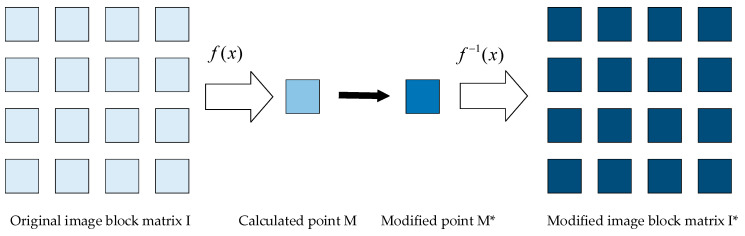
The process of changing the unit module.

The customized 2D barcode uses a unit image block as the fundamental encoding unit. Additionally, the amount of change in the image block is as small as possible, so that it can ensure the decoding robustness and improve the perceptual quality of embedding images. The main idea of the fundamental encoding unit is to use an image block of k×k pixels as a whole for calculation, instead of the traditional information hiding algorithm that uses a single pixel as the operation object. When encoding and decoding bits, the idea of utilizing the whole image block is adopted. First, the image block of the k×k pixels is represented by a point *M* by the f(x) operation, then the point *M* is encoded according to the coding rules to make it point M*, and finally the encoded image block is calculated according to the change of point *M*. Figure 3 illustrates the process of changing the unit module by image block coding.

**Figure 4:**
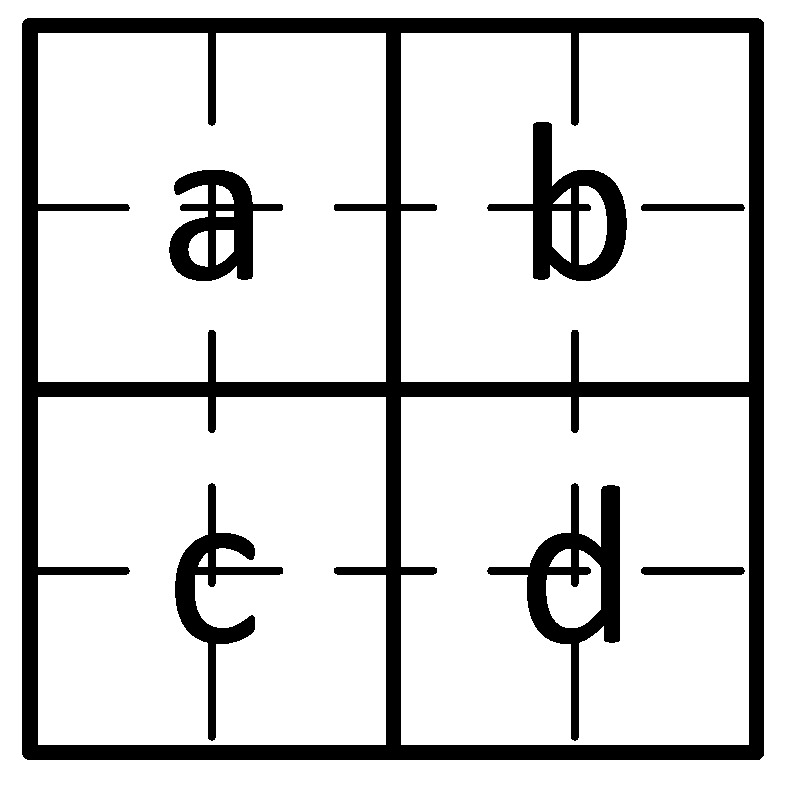
The fundamental encoding unit.

As shown in Figure 4, the fundamental encoding unit consists of four basic image blocks *a*, *b*, *c*, and *d*. For the convenience of description, we assume that the judgment graphic size is 4×4 pixels, which is the smallest size, so the size of each basic image block is 2×2 pixels.

In practical application, the size of each judgment graphic should be adjusted, following the requirements of robustness and visibility flexibly. The larger the judgment graph, the stronger the robustness, but the worse the visibility, and vice versa. Therefore, the setting of the judgment graphic should not be less than 4×4 pixels, which is increased by a multiple of 2. The encoding is performed according to the average pixel value of four image blocks, which assumes the average gray values of four basic image blocks are $M_{a}$, $M_{b}$, $M_{c}$, and $M_{d}$, respectively. In this study, we determine the encoding of “0” or “1” by the size sorting relationship between these values. It is further considered that the comparison of four values could be divided into two or more levels. The more levels of subdivisions, the more detailed size judgment could be achieved. However, it also brings challenges to the decoding process, because the greater the levels of subdivisions, the fewer pixels per block corresponding to the number of pixels. As a result, each block’s average value is more susceptible to the environment and the effects of various scaling. Selecting only two levels is more destructive to the image and unfriendly to human eyes, while four levels or more will have a more significant impact on the decoding robustness. Hence, we choose three levels of comparison after judicious consideration.

The strategy adopted was to calculate the grayscale values of four image blocks into three standard grayscale values, by setting $M_{bc}=\frac{M_{b}+M_{c}}{2}$. Then, the sequence $\left\{M_{a},M_{bc},M_{d}\right\}$ was sorted in the ascending order, and the encoding rules were defined as follows:

(1) $$Bit=\{\begin{array}{l} 0,\;M_{a}\;is\;in\;the\;\sec ond\;position\\ 1,\;M_{a}\;is\;in\;the\;remaining\;position \end{array}$$

After determining the size relationship between $\left\{M_{a},M_{bc},M_{d}\right\}$, the difference between them was found to be higher than a fixed value represented by δ, to ensure decoding robustness. δ could be adjusted as needed, to balance the aesthetic of the picture and the decoding robustness.

*Case 1: The sequence* $\left\{M_{a},M_{bc},M_{d}\right\}$ *is sorted correctly.*

1. Bit=0, $M_{a}$ is in the second position. Suppose $M_{bc}$ and $M_{d}$ are in the first and third place, respectively, and the value of $M_{a}$ remains unchanged. If the differences between each pair of $M_{a}$, $M_{bc}$, and $M_{d}$ are no less than the threshold δ, the value remains unchanged. Otherwise, the former is added by δ, whilst the latter is reduced by δ to enlarge the differences to be no less than δ. This process could be expressed as:
  (2) $$M_{a}^{*}=M_{a}$$
  (3) $$M_{bc}^{*}=\left\{ \begin{array}{l} M_{bc},\;if\;M_{bc}-M_{a}\geq \delta \\ M_{bc}+\delta ,\;if\;M_{bc}-M_{a}<\delta \end{array}\right.$$
  (4) $$M_{d}^{*}=\left\{ \begin{array}{l} M_{d},\;if\;M_{d}-M_{a}\geq \delta \\ M_{a}-\delta ,\;if\;M_{d}-M_{a}<\delta \end{array}\right.$$
2. Bit=1, $M_{a}$ is in the remaining position. Suppose $M_{a}$, $M_{bc}$, and $M_{d}$ are in the first, second, and third places, respectively. Similar to the first case, the value of $M_{d}$ remains unchanged, and we need to ensure that the differences between $M_{a}$ and $M_{bc}$, and $M_{a}$ and $M_{d}$ are no less than the threshold δ. If the differences between them meet the requirements, the original value remains unchanged. Otherwise, the first position subtracts the corresponding second and third position to get a new value, respectively, ensuring that the differences are no less than the threshold δ. This process could be expressed as:
  (5) $$M_{a}^{*}=M_{a}$$
  (6) $$M_{bc}^{*}=\left\{ \begin{array}{l} M_{bc},\;if\;M_{a}-M_{bc}\geq \delta \\ M_{a}-\delta ,\;if\;M_{a}-M_{bc}<\delta \end{array}\right.$$
  (7) $$M_{d}^{*}=\left\{ \begin{array}{l} M_{d},\;if\;M_{a}-M_{d}\geq \delta \\ M_{a}-\delta ,\;if\;M_{a}-M_{d}<\delta \end{array}\right.\;$$

*Case 2: The sequence* $\left\{M_{a},M_{bc},M_{d}\right\}$ *is sorted incorrectly.*

1. Bit=0, $M_{a}$ is in the first or the third position. Suppose $M_{a}$, $M_{bc}$, and $M_{d}$ are in the first, second, and third places, respectively. The correct order of adjustment is that $M_{bc}$ is ranked first, $M_{a}$ is second, and $M_{d}$ is third. In addition, we utilize the median exchange method to calculate the amount of value change to reduce the impact on the aesthetics of pictures due to value change. Namely, $M_{a}^{*}$ is equivalent to the average of $M_{a}$ and $M_{bc}$, and $M_{bc}^{*}$ is $M_{a}$ changed plus the threshold δ. If the difference between the values of $M_{d}$ and $M_{a}^{*}$ is no less than the threshold δ, the original value remains unchanged. Conversely, $M_{a}$ subtracts the threshold δ to get a new value. This process could be expressed as:
  (8) $$M_{a}^{*}=\frac{M_{a}+M_{bc}}{2}$$
  (9) $$M_{bc}^{*}=M_{a}^{*}+\delta$$
  (10) $$M_{d}^{*}=\left\{ \begin{array}{l} M_{d},\;if\;M_{a}^{*}-M_{d}\geq \delta \\ M_{a}-\delta ,\;if\;M_{a}^{*}-M_{d}<\delta \end{array}\right.$$
2. Bit=1, $M_{a}$ is in the second position. Suppose $M_{bc}$ is in the first place, and $M_{d}$ is in the third. To reduce the impact on the aesthetics of pictures due to value change, $M_{a}$ exchanges the position with the closer distance. Assuming that $M_{bc}$ is close to $M_{a}$, which means that $M_{bc}-M_{a}\leq M_{a}-M_{d}$. Then $M_{bc}^{*}$ is equal to the average of $M_{a}$ and $M_{bc}$, and $M_{a}^{*}$ is $M_{bc}$ after changes, plus the threshold δ. If the difference between the values of $M_{d}$ and $M_{a}$ after being changed is no less than the threshold δ, the original value remains unchanged. Conversely, $M_{a}$ subtracts the threshold δ to get a new value. This process could be expressed as:
  (11) $$M_{bc}^{*}=\frac{M_{a}+M_{bc}}{2}$$
  (12) $$M_{a}^{*}=M_{bc}^{*}+\delta$$
  (13) $$M_{d}^{*}=\left\{ \begin{array}{l} M_{d},\;if\;M_{a}^{*}-M_{d}\geq \delta \\ M_{a}-\delta ,\;if\;M_{a}^{*}-M_{d}<\delta \end{array}\right.$$

After completing the above process, a zeroing process is applied on the changed value of each block to maintain the consistency of the mean of the changed image block and the original image block. Denote the changes of $M_{a}$, $M_{bc}$ and $M_{d}$ as Δa, Δbc and Δd, respectively, we have:

(14) $$\begin{array}{l} \Delta a=M_{a}^{*}-M_{a}\\ \Delta bc=M_{bc}^{*}-M_{bc}\\ \Delta d=M_{d}^{*}-M_{d} \end{array}$$

During the zeroing process, Δbc represents two image blocks, while Δa and Δd represent one image block, respectively. The sum of the statistical change amounts is Δm=Δa+2×Δbc+Δd, and the change of each block is the difference between the first change and the statistical change, expressed as $\Delta a^{*}$, $\Delta bc^{*}$, and $\Delta d^{*}$.

(15) $$\begin{array}{l} \Delta a^{*}=\Delta a-\Delta m\\ \Delta bc^{*}=\Delta bc-\Delta m\\ \Delta d^{*}=\Delta d-\Delta m \end{array}$$

The changes are diffused to the corresponding block, which means the pixel value of the corresponding block adds the corresponding change. The process is as follows:

(16) $$I^{*}(x,y)=I(x,y)+\left\{ \begin{array}{l} \Delta a^{*},\;xy\in a\\ \Delta bc^{*},\;xy\in b\;or\;c\\ \Delta d^{*},\;xy\in d \end{array}\right.$$

where I(x,y) denotes the pixel value of the corresponding block.

After the above process, the Y channel of the original image is completely encoded. Then, it is combined with remaining unchanged channels to form a color image carrying the encoded information. Finally, the detection area is increased to form a customized 2D barcode, according to the size of the customized 2D barcode determined by the encoding information.

## 3. The Recognition Algorithm of Customized 2D Barcodes Sensing

To detect the customized two-dimensional (2D) barcodes, the Data Matrix code detection method is used for reference. We chose the ZXing open-source kit [32] to detect customized 2D barcodes for the sake of stability and universality. Moreover, in the recognition stage, only the pixels of each image block center part are employed in the traditional 2D barcode sampling process. While customized 2D barcodes must sample the entire image block, it is necessary to make some optimization of sampling to achieve the requisite recognition accuracy.

**Figure 5:**
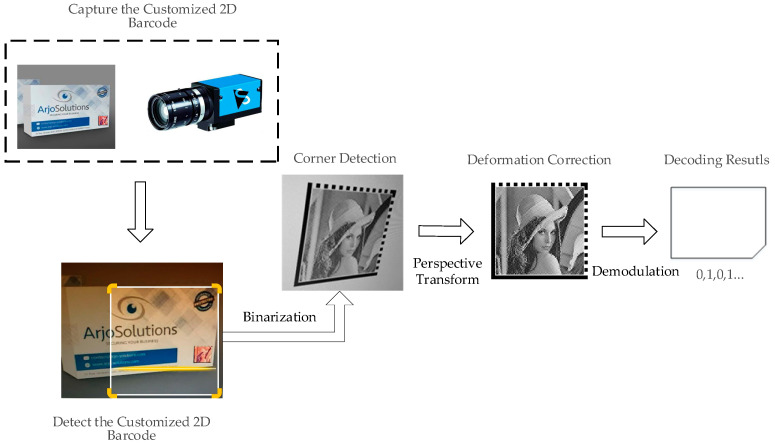
The recognition procedure of customized 2D barcodes sensing.

The images acquired by a camera are always scaled and rotated in one or more directions. Therefore, the relevant method-like perspective transform should be used for correction to achieve the accuracy of recognition. The recognition procedure of the customized 2D barcode sensing is shown in Figure 5. First, the captured customized 2D barcode is converted to a grayscale image, and only the information of the Y channel is extracted. Then, the image performs the binarization to determine the effective regions by detecting the corner points using the ZXing library. The perspective transform step is the correction of the binarized image to facilitate the decoding process. Based on the grid-decoded process, the correct ZXing is obtained. Finally, the input information is reconstructed by the bitstream.

### 3.1. Corner Detection

**Figure 6:**
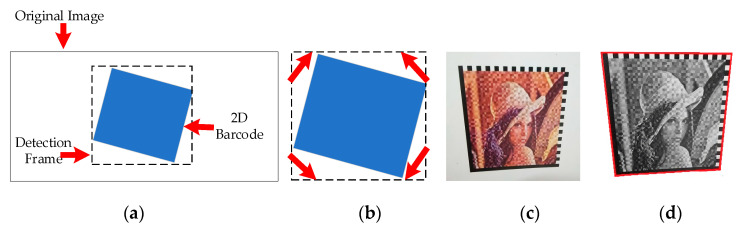
The schematic diagram of corner detection. (**a**) The process of corner detection; (**b**) determining the vertices of the four corners of the 2D barcode image; (**c**) capturing the customized 2D barcode image; and (**d**) performing position detection.

Figure 6a illustrates the process of corner detection. At the beginning of the detection, a 30×30 rectangle is framed in the middle of the image, and then the rectangle is advanced toward the four sides in turn. During every advancement process, it determines if there are black dots on the sides of the rectangle, and the iteration stops scanning when there are no black dots on each side of the rectangle. As shown in Figure 6a, the blue area is a 2D barcode image, and the dashed frame is a frame containing the 2D barcode area.

The 45° straight line is used to determine the vertices of the four corners of the 2D barcode image. As shown in Figure 6b, the red arrows indicate the direction of detection, and scanning is stopped when a black dot is found. If there are no black dots, it is inevitable that the scan direction should be adjusted until detecting the black dots. The key part of the decoding process is to determine the locator of the 2D barcode image. Since the position patterns of the visualized 2D barcode are two ‘L’ patterns, after determining the four vertices, one L-shape is a solid line, and the other L-shape is a dashed line. Therefore, we adopt the strategy based on the finder pattern of the customized 2D barcode, to detect the number of black-and-white transitions. Additionally, the method of drawing straight lines is based on the Bresenham’s algorithm [26]. The solid line has fewer black and white transitions, while the virtual line has more black and white transitions. Therefore, the intersection of the two ‘L’ patterns could be found, then the intersection points of the lower left corner and the upper right corner could quickly be calculated, and finally the position of the other two points according to these two points could be determined. The subsequent step is to obtain the version and the shape of the 2D barcode, then count the number of black-and-white transformations on the edge of the dashed line. Since the data modules of the visualized 2D barcode are even numbers, if the number of transformations is odd, error correction is required. After counting the number of modules, we can know the width of each module, and how many modules are on each side. Thus, the version information of the 2D barcode could be obtained. We performed position detection on the visualized 2D barcode, and connected four points found with red lines. This process is shown in Figure 6c,d; Figure 6c shows the captured visualized 2D barcode image, and Figure 6d is the image after position detection.

### 3.2. Deformation Correction of the Customized 2D Barcode

During the acquisition of the customized 2D barcode, there are deformations, including oblique deformation and perspective deformation. Since the visualized 2D barcodes sample the entire image block, the deformation effect is more severe. Therefore, it is necessary to correct the image to overcome the adverse effects of geometric change, before identifying the visualized 2D barcode. The image correction includes the spatial transformation of pixels and the grayscale interpolation of pixel coordinates. Perspective transform achieves the correction of the distortion image through a 30×30 matrix. The formula is shown in Equation (17):

(17) $$\left[\begin{array}{c} x^{*}\\ y^{*}\\ w^{*} \end{array}\right]=\left[\begin{array}{ccc} a_{11} & a_{12} & a_{13}\\ a_{21} & a_{22} & a_{23}\\ a_{31} & a_{32} & a_{33} \end{array}\right]\left[\begin{array}{c} u\\ v\\ w \end{array}\right]$$

where $\left[\begin{array}{cc} u & v \end{array}\right]$ is the coordinate in two-dimensional distortion space, while $\left[\begin{array}{cc} x & y \end{array}\right]$ is the corresponding point coordinate in two-dimensional correction space. Then, its homogeneous coordinate is $\left[\begin{array}{cc} x=\frac{x^{*}}{w^{*}} & y=\frac{y*}{w^{*}} \end{array}\right]$, where w represents the ratio between the two coordinate systems. The relationship between $\left[\begin{array}{cc} x & y \end{array}\right]$ and $\left[\begin{array}{cc} u & v \end{array}\right]$ is as follows:

(18) $$x=a_{11}u+a_{12}v+a_{13}-a_{31}u\times x-a_{32}v\times x$$

(19) $$y=a_{21}u+a_{22}v+a_{23}-a_{31}u\times y-a_{32}v\times y$$

Four vertices coordinates of a corrected 2D barcode are denoted as $\left(x_{0},y_{0}\right)$, $\left(x_{1},y_{1}\right)$, $\left(x_{2},y_{2}\right)$, $\left(x_{3},y_{3}\right)$. According to its square structure, the general settings are (0,0), (0,L), (L,0), (L,L), and L are constants, corresponding to the upper left corner, upper right corner, lower right corner, and lower left corner of the rectangle. Assuming that the corresponding four vertices of the distorted 2D barcode are $\left(x_{0}^{*},y_{0}^{*}\right)$, $\left(x_{1}^{*},y_{1}^{*}\right)$, $\left(x_{2}^{*},y_{2}^{*}\right)$, $\left(x_{3}^{*},y_{3}^{*}\right)$, and the scale factor is set as $a_{33}=1$. By knowing the coordinates of these eight points, a perspective transformation matrix could be calculated. Through the matrix, the pixels in the distorted customized 2D barcode area could be mapped to a square, which is established according to the prior barcode knowledge. However, the calculated value would have non-integers, therefore, it is necessary to use interpolation to take the integer. In practical applications, the accuracy requirements are not particularly high, while the time required for decoding is short. The proposed encoding method is robust against scaling, because it is based on the unit image block. Therefore, bilinear interpolation is selected for the gray value correction. It is assumed that f(x,y) is the gray value calculated at the coordinate point (x,y) using the following bilinear interpolation:

(20) $$f(x,y)=a_{1}+a_{2}x+a_{3}y+a_{4}$$

The coordinates and gray values of the four adjacent points around the sampling point (x,y) are known. To facilitate processing, the coordinates of these four points are normalized to correspond to the four vertices of the square, with standardized values of f(0,0), f(1,0), f(0,1), and f(1,1). The values of these four points determine f(x,y). It does not matter if first interpolate is in the x or *y* direction. In this study, we first interpolate the upper two vertices linearly, to get:

(21) f(x,0)=f(0,0)+x[f(1,0)−f(0,0)]

Then, we interpolate the lower two vertices linearly to get

(22) f(x,1)=f(0,1)+x[f(1,1)−f(0,1)]

Finally, we interpolate linearly in the vertical direction to get

(23) f(x,y)=f(x,0)+y[f(x,1)−f(x,0)]

Combining the above equations, we can infer:

(24) f(x,y)=x[f(1,0)−f(0,0)]+y[f(0,1)−f(0,0)]+xy[f(1,1)+f(0,0)−f(0,1)−f(1,0)]

**Figure 7:**
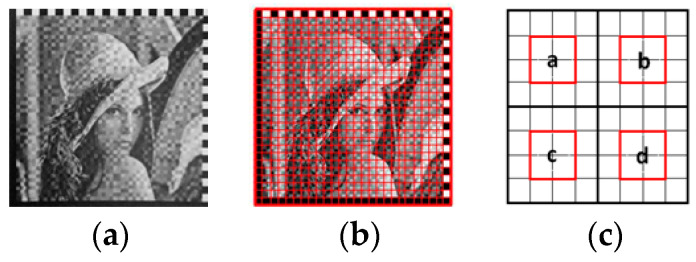
Corrected grayscale image and decoded grids. (**a**) Corrected grayscale image. (**b**) Corrected grayscale image with decoded grids. (**c**) The scheme of decoded grids.

After perspective transform and linear transform, the original grayscale image is well restored, as shown in Figure 7a,b.

### 3.3. Customized 2D Barcode Decoding

First, we resized the image to make every module correspond to a decoding unit. During the module decoding stage, each image block is resized into an 8×8 image block, which is the minimum number of pixels required by the proposed algorithm. Similar to the encoding stage, the decoding stage is the inverse process of the encoding stage, which is to count the average of the pixels of each module, and then determine the encoded bit by determining the size. However, due to the interference of various factors during image acquisition, such as the effects of lighting, distortion, and noise, especially in the edges, it is more susceptible to interference by scaling factors. Although the unit image block algorithm has a good ability to resist interference from scaling factors, it is better to optimize to achieve higher accuracy. Hence, we search the sample point in the captured 8×8 image block again. As shown in Figure 7c, we define the four areas *a*, *b*, *c*, and *d* as parts in the red box, and in each area only 4 pixels in the center are considered to prevent the blurring caused by the scaling factors. Furthermore, it preserves good decoding robustness in the case where the positioning is not very precise. Similar to the encoding stage, the average gray levels of the four basic image in Figure 7c are $M_{a}$, $M_{b}$, $M_{c}$, and $M_{d}$, and $M_{bc}=\frac{M_{b}+M_{c}}{2}$. The value of Bit can be obtained according to the sorting result of the sequence $\left\{M_{a},M_{bc},M_{d}\right\}$, as follows:

(25) $$Bit=\{\begin{array}{l} 0,\;M_{a}\;is\;in\;the\;\sec ond\;position\\ 1,\;M_{a}\;is\;in\;the\;remaining\;position \end{array}$$

We can decode each image block in turn and obtain an N×N matrix, formed sequentially. The modules are then rearranged according to the version information. Finally, error correction and data decoding can be performed to restore the original encoding information.

## 4. Experimental Results and Analysis

The encoding information is embedded into the carrier image, resulting in a decrease of image quality with the embedding, and different images have different degrees of damage. It is necessary to introduce some objective indicators to quantify the degree of damage to the carrier image after embedding the information. Moreover, it is necessary to evaluate the encoding and decoding speed to determine whether the proposed 2D barcode has the functional characteristics of the traditional 2D barcode and to check if it meets the requirements of fast recognition for anti-counterfeiting verification.

### 4.1. Perceptual Quality and Encoding Time

**Figure 8:**
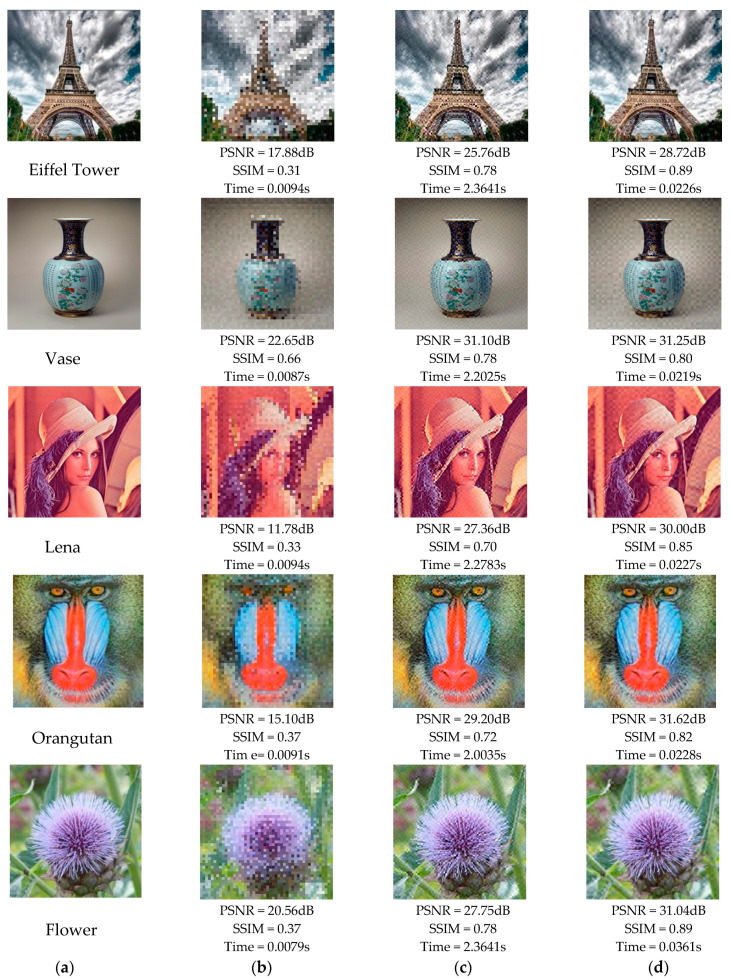
The perceptual quality and encoding time comparisons of different aesthetic 2D barcodes. (**a**) Carrier images, (**b**) SRA codes [28] with threshold δ=10, (**c**) RA codes [31] with threshold δ=10, and (**d**) our codes with threshold δ=10.

**Figure 9:**
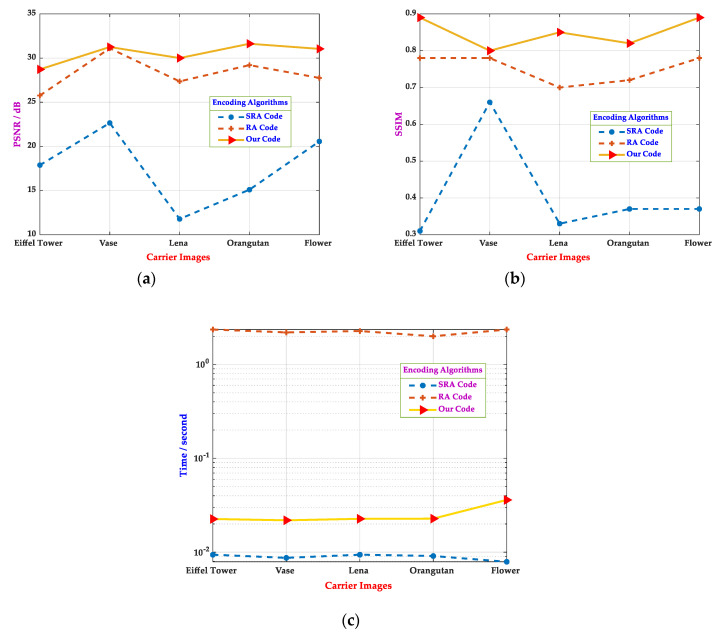
The perceptual quality and encoding time comparisons of aesthetic 2D barcodes with different carrier images. (**a**) The PSNR values of different carrier images when threshold δ=10. (**b**) The SSIM values of different carrier images when threshold δ=10. (**c**) The encoding time of different carrier images when threshold δ=10.

In this work, the objective evaluation criteria selected were Peak Signal to Noise Ratio (PSNR) and Structural Similarity (SSIM) [33], the larger their values, the better the encoded image quality. Moreover, several encoding methods were compared in encoding time, which could reflect the practical characteristics of various algorithms. By combining various indicators, the characteristics of each method could be more analyzed objectively. In order to reflect the fairness of the experiments, we ensured the coherence of the test picture size and the encoding module size. As shown in Figure 8, five images with a resolution of 288 pixels×288 pixels were used as carrier pictures, whose contents were the Eiffel Tower, Vase, Lena, Orangutan, and Flower. This set of pictures was very demonstrative, including different backgrounds and different things, and relatively rich textures. Based on the consideration of image data capacity and picture size, each sub-module of the carrier image was set as 24 pixels×24 pixels. The testing results of the different encoded pictures are shown in Figure 9:

The previously encoded carrier image was observed under the same threshold, where the T value in the RA code corresponded to the threshold δ in this study. For example, the T=0.75 in the RA code, the threshold δ=10 in the remaining methods. As shown in Figure 9a,b, the experimental results demonstrated that both PSNR and SSIM of the customized 2D barcode were higher than that of the RA code, and both PSNR and SSIM of the SRA code were lower than that of the RA code. It meant that the customized 2D barcode had a better perceptual quality than the RA code and the SRA code. Moreover, under the same conditions, the results in Figure 9c illustrate that the encoding efficiency of the customized 2D barcode was much higher than that of the RA code, and only slightly lower than that of the SRA code. However, as shown in Figure 8b, the SRA code performed poorly on the quality of the encoded image. The experiments in Figure 8 and Figure 9 showed that the customized 2D barcode was superior to other encoding schemes in encoding picture quality and had a better encoding time. It is known that as the threshold increases, the quality of the picture and the PSNR and SSIM indicators decrease. Additionally, the threshold is related to the decoding robustness. When the threshold increases, the bearing capacity of the carrier image in terms of the effects of distortion, compression, and other factors improve accordingly, which could be applied to different complex scenarios.

In Section 4.2, the decoding robustness of various aesthetic 2D barcodes was evaluated under the same perceptual quality.

### 4.2. Decoding Robustness in Sensing Recognition

In this section, the digital pictures and printed photos were selected for the experimental tests, in which the photo size was 4 × 4 cm^2^. The experimental configurations are shown as follows:

- Rendering
  - Printing: HP Color LaserJet CP5225dn in 600×600 dpi on paper with 160 g/m^2^;
  - Displaying: The HP Zhan *X* with retina display at 200 ppi;
- Carrier images: Five images in Figure 8;
- Barcode design: SRA Code, RA Code and customized 2D barcode;
- Module design: 24×24 modules;
- Angle: Perspective angle at ±20°;
- Capture angle: Rotate in 0–180°;
- Capture distance: Around 7 cm;
- Decontamination: Blackening random areas of images;
- Light intensity: Indoor lighting with 300–350 lux.

**Figure 10:**
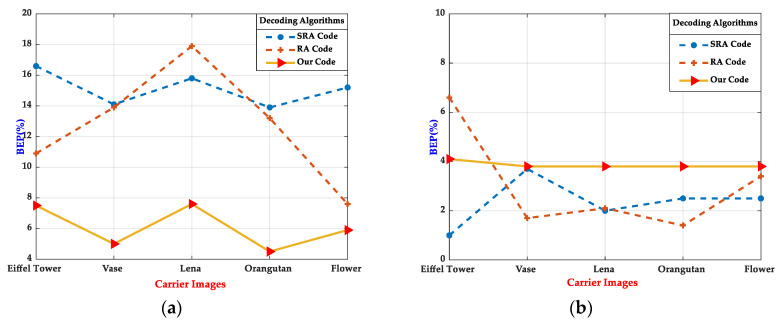
Decoding robustness comparisons in terms of demodulation bit error probability (BEP) in printed photos taken in actual scenes (**a**) and digital pictures (**b**).

Consequently, we referred to the above conditions to check the performance of the encoding schemes, and detection indicators were the bit error probability (BEP) and the decoding time. The choice of angle and illumination parameters was derived from the observation and summary of practical applications in life. For example, when δ=20, the sensing recognition error rate of the three visualized 2D codes are shown in Figure 10, including printed on A4 paper (see Figure 10a) and a digital version (see Figure 10b), and size was 4×4 (cm^2^). The experimental results illustrate that the proposed customized 2D barcode achieved the lowest demodulation BEPs, across all printed photos taken in actual scenes, and belonged to the same levels as the SRA Code and the RA Code in the digital version.

In terms of decoding time (see Table 1), we selected five types of pictures as experimental objects, and under each type, five pictures were collected to calculate the mean. The results showed that the customized 2D barcode and RA Code were faster than the SRA Code in decoding time, and the customized 2D barcode had a slight advantage than the RA Code. Moreover, the same trend was also observed in the printed photos.

The analysis of Figure 10 and Table 1 revealed that the customized 2D barcode was more robust in the actual environment, which could successfully and rapidly decode and restore the original content. Additionally, we used multiple images for the experiments to demonstrate the universality of the approach, where the resolution and content of the encoded image were the same. After experimental testing, the results reached the same level in various indicators, which indicated that the customized 2D barcode had an excellent performance in practicality.

### 4.3. Experimental Results of Varying Thresholds

It is known that the change of the threshold affected the encoded image quality. If the threshold was too large, the embedded image would be severely damaged, resulting in poor aesthetic appearance, which indicated that the encoding scheme was worthless in practical applications. Additionally, compared to the traditional 2D barcode, the superiority of the visual 2D barcode lay in its aesthetic appearance, which could obtain part of the information directly through human eyes. Based on this feature, we re-examined the experimental results of encoding and decoding and found that the visibility of the SRA code was much lower than that of the customized 2D barcode and the RA code, while they showed stronger decoding robustness.

**Figure 11:**
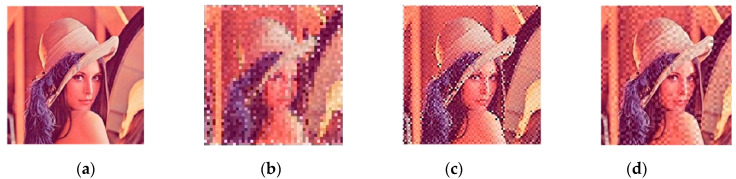
The perceptual quality of aesthetic 2D barcodes with Lena: (**a**) Carrier image, (**b**) SRA code [28] with the threshold *δ* = 20, (**c**) RA code [31] with *δ* = 20, and (**d**) our code with *δ* = 20.

**Figure 12:**
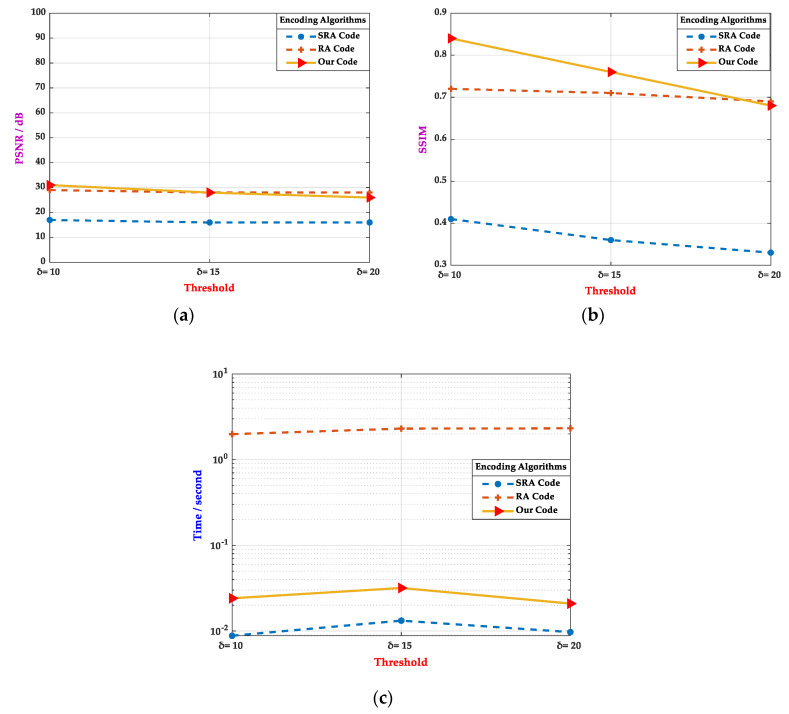
The perceptual quality and time consuming of various encoding algorithms under different thresholds. (**a**) The PSNR of various encoding algorithms under different thresholds. (**b**) The SSIM of various encoding algorithms under different thresholds. (**c**) The time consumed by various encoding algorithms under different thresholds.

As shown in Figure 11, an example of the picture-embedding 2D barcodes when δ=20, the RA code and the FA code, could maintain certain perception quality, while the SRA performed poorly in observability. Comprehensive analysis showed that the customized 2D barcode could well realize the original intention of the design. Under the premise of ensuring image visibility, the encoding experiments were mainly carried out with thresholds 10, 15, and 20. The impact indices of different threshold-coded images were mainly compared using SSIM, PSNR, and the encoding time. Statistical analyses were performed on the 200 samples of SSIM, PSNR, and the average time consumed. The results are shown in Figure 12.

As can be seen in Figure 12a,b, the customized 2D barcode had advantages in PSNR and SSIM, when the thresholds were 10 and 15, and were close to the proposed 2D barcode when the threshold was 20, which was far superior to the SRA code. Additionally, as shown in Figure 12c, in terms of encoding time, the customized 2D barcode belonged to the same order of magnitude as the SRA code, with a time of about 30 milliseconds, which was far better than the RA code, whose encoding time took about 2 s.

Considering the above experimental results, the customized 2D barcode is leading in most projects. Through a comprehensive analysis from the characteristics of the human eye and various evaluation indicators, the customized 2D barcode had the excellence of a high encoding rate and a better perceptual quality, which is more in line with the visual feeling of human eyes. Furthermore, the experimental results illustrate that the customized 2D barcode could maintain a high encoding speed.

## 5. Discussion

The main factor driving us to conduct this research was how to design a better image identification, which could maximize the role of image identification as the perception portal of the IoT. Through the design of encoding and decoding methods and experimental verification, we designed a customized visual 2D barcode identification with excellent characteristics. The anti-counterfeiting of the customized 2D barcode was manifested in two aspects. On the one hand, it could be directly seen visually and reflect the difference of the product. Enterprises can customize the design of two-dimensional barcodes according to their needs, which could ensure high visual security. On the other hand, the generated customized 2D barcode is a closed source code. Unless the encoding algorithm is cracked, pure copying is meaningless. Moreover, the customized 2D barcode could easily be combined with the existing image anti-counterfeiting technology. For instance, designing unique texture features in certain color channels would realize more secret and safe anti-counterfeiting.

As mentioned in the previous introduction, there is a unique traceability anti-counterfeiting barcode corresponding to the product’s traceability process. As the carrier of the traceability system, the traceability anti-counterfeiting barcode records the related information of the whole process, in order to realize the traceability of products. The proposed customized 2D barcode had the functions of traditional 2D codes, while achieving visual security. The format and length of the encoded content were not specified. Therefore, it could be encoded, combined with related standards, to achieve one code for one object, which was not limited to the international GS1 Digital Link standard, as well as the Chinese Ecode IoT identification standards. When the enterprise uses traceability anti-counterfeiting barcode, the customized 2D barcode could be encoded according to the above standards, and the carrier images and encoding contents could be customized according to the requirements.

## 6. Conclusions

In this study, a customized 2D barcode with fast encoding for smart IoT applications was proposed, based on information hiding technology, which obtained the ranking results of each image basic encoding module to define the corresponding image bit value. Then, it achieved a fast encoding and a better aesthetic appearance. After optimizing the sampling network of sensing recognition, the decoding time was as short as possible, while ensuring decoding robustness. The proposed customized 2D barcode and sensing recognition scheme showed an excellent performance in various authentic environments, compared to other state-of-the-art approaches, which could meet the practical requirements. Intensive experiments illustrated that a customized 2D barcode has a strong practical value in visual anti-counterfeiting. Additionally, the proposed 2D barcode with a sensing recognition scheme could be designed as a custom, closed-source system for specialized fields, which could significantly improve the security of data information and have a great potential in the IoT anti-counterfeiting traceability application.

## Figures and Tables

**Table 1:** Decoding robustness comparisons in terms of decoding time. The size of the printed picture was 4×4 cm^2^ and the threshold value was 20.

Embedded Image	SRA Code [28]		RA Code [31]		Our Code	
	Print (Time/ms)	Digital (Time/ms)	Print (Time/ms)	Digital (Time/ms)	Print (Time/ms)	Digital (Time/ms)
Eiffel Tower	95.1	81.4	4.9	4.2	3.8	3.6
Vase	90.3	81.5	4.6	4.4	3.6	3.5
Lena	89.7	83.1	4.8	4.1	3.7	3.7
Orangutan	81.3	80.4	4.6	3.9	3.9	3.8
Flower	93.3	89.1	4.8	3.8	3.5	3.4
